# Supplementary material for: COVID-19 vaccine hesitancy and refusal and associated factors in an adult population in Saskatchewan, Canada: Evidence from predictive modelling
Source: PLoS One. 2021 Nov 12;16(11):e0259513. doi: 10.1371/journal.pone.0259513 (PMC8589208; doi:10.1371/journal.pone.0259513)
Supplement: S1 Box — (DOCX) [file pone.0259513.s001.docx]

**S1 Box: Description of independent variables**

Sociodemographic variables included age, gender, employment status, highest educational attainment, place of residence, immigration status, Indigenous status, and co-residence. The variables on risk behaviours for coronavirus included level of exposure at places visited, number and duration of contacts, level of exposure at work, current work situation, financial stability, places visited in the past 7 days for grocery shopping, household population density, and recent travels outside Saskatchewan. The variables on mitigating factors against COVID-19 risk included facemask wearing, social distancing, current perceived health status, and being tested for COVID-19. Community perception variables included opinions about the adequacy of government measures in combating the pandemic, community adherence to public health measures, threat of COVID-19 to community, likelihood of contracting the virus with current lifestyle, fears of illness/death after getting COVID-19, and concerns about spreading infections after being infected.

The risk levels of exposure at places visited were measured by asking the respondents places visited in the past 7 days before the survey. Based on possibility of physical distancing, public outdoor space (such as park, trail, and nature) and homestay were assigned as low risk. Moderate risk gatherings were indoor store, drop-in centres, and indoor event venues (such as hotel, community centre, worship centre, banquet hall, and meeting room), and high-risk places were categorized as gym/fitness centre, restaurant/bar/pub/night club, casino, someone else’s homes, personal/health care services (hair salon/barber, message therapist, chiropractor etc.), school/daycare and long-term care home or seniors’ residence. Using tertile classification, risk level of contacts was constructed from a composite variable of two independent but related variables on the number and duration of contacts. To assess level of exposure to COVID-19 at work, respondents were asked seven items relating to chance of contracting the virus through volunteering or working outside their homes. The response scale was recoded as “not exposed or low risk” (0), “moderate risk” (1-3) and “high risk” (4-6). Also, the current work/volunteering situation was described as low risk if respondents did not work/volunteer outside their homes or worked/volunteered outside homes without interacting with people outside their households, moderate risk if worked outside their homes in an indoor setting with co-workers who did not interact with the public, and high risk if worked outside their homes primarily in an indoor setting with interaction with different people. The risk level of COVID exposure for grocery shopping was calculated by assigning “did not currently shop for groceries” low risk, “visited physical stores at most once a month” moderate risk, and “visited physical stores everyday or a couple of times a week” high risk. Perceived financial security was measured one item scale: “Do you feel financially secure right now?” (absolutely not secure, somewhat not secure, a little bit not secure, a little bit secure, somewhat secure, and absolutely secure). The current perceived health status was assessed by asking the respondents, “Compared to other people your age, how would you rate your current health?” (excellent, very good, good, fair and poor).
