# Supplementary material for: COVID-19 vaccine hesitancy and refusal and associated factors in an adult population in Saskatchewan, Canada: Evidence from predictive modelling
Source: PLoS One. 2021 Nov 12;16(11):e0259513. doi: 10.1371/journal.pone.0259513 (PMC8589208; doi:10.1371/journal.pone.0259513)
Supplement: S1 File — (DOCX) [file pone.0259513.s002.docx]

**S1 File**

**Consent note**

**This research project has been approved on ethical grounds by the University of Saskatchewan Research** **Ethics Board (Beh-1971).**

Any questions regarding your rights as a participant may be addressed to that committee through the Research Ethics Office ethics.office@usask.ca (306) 966-2975. Out-of-town participants may call toll free (888) 966-2975.

**Participation is strictly voluntary.**In order to complete this survey, you must provide a response to each question. If you are unable or unwilling to provide a response to a question or to complete the questionnaire, you may withdraw at any time by closing your internet browser. Once you have submitted the survey, you are no longer able to withdraw your data as your responses are anonymous and we will not be able to identify the data specifically to you. Your responses are confidential. All data provided by SSRL to the researchers will be treated confidentially and results will be reported anonymously and in an aggregated or summarized form.

Data will be stored in on the University of Saskatchewan’s password-protected, acccess-managed, enterprise-class DATASTORE using industry best practices for security. Access will be managed by the Principal Investigator and only made available to designated research team members. Data will be stored on the DATASTORE for 5 years post publication, then completely, securely, and permanently deleted.

Answering questions about risk of contracting or spreading COVID-19 and increasing your understanding of COVID-19 prevention and transmission may invoke emotional or psychological responses, including fear, anxiety, stress, feelings of powerlessness, vulnerability, hypochondria, feeling overwhelmed, and/or worry.

If you think you may have been exposed to COVID-19 or have symptoms, please complete the Government of Saskatchewan’s COVID-19 Self-Assessment or call the HealthLine at 811.

For information on how to get tested for COVID-19 in Saskatchewan, click here or call the HealthLine at 811.

If you are experiencing any psychological or emotional responses to this questionnaire, please see the Government of Saskatchewan’s Mental Health page for information on supports and resources.

**More COVID-19 Information**

Government of Saskatchewan: <https://www.saskatchewan.ca/government/health-care-administration-and-providerresources/treatment-procedures-and-guidelines/emerging-public-health-issues/2019-novel-coronavirus/resources>

Government of Canada: <https://www.canada.ca/en/public-health/services/diseases/coronavirus-disease-covid-19.html>

**By selecting “next” and completing this questionnaire, your free and informed consent is implied and indicates** **you understand the above conditions to participate in this study.**

Please consider printing or saving this page for your records.
